# English Dentistry and the Chemists

**Published:** 1878-08

**Authors:** 


### EDITORIAL, ETC.

---

*English Dentistry and the Chemists.*—In the *Chemist and Druggist* is an extended report of the visitation by a delegation of druggists to the "Promoters of the Dental Practitioner's Bill," with a view of obtaining some modification of its terms so far as they relate to the practice of dentistry by druggists. The effect of the bill as drafted, it was claimed by the delegation would be "to prevent all those chemists and druggists who were at that time practising dentistry from continuing to do so, or at all events from recovering any fee or charge, in any Court, for the performance of any dental operation, unless they obtained their registration under the Act, for which there seemed to be no provision in the Bill ; on the contrary, they were by the fifth clause debarred from registration, not being "licentiates in dental surgery or dentistry of any Royal College of Surgeons in the United Kingdom, or of the Faculty of Physicians and Surgeons of Glasgow; or at the passing of that Act *bona fide* engaged in the practice of dentistry, either separately or in conjunction with the practice of medicine or surgery." This prohibition would be very severely felt by many of Her Majesty's poorer subjects throughout the kingdom, and it would be a source of great inconvenience to all classes of the public residing in country districts where no professional dentist was to be found."

Apparently the framer of the bill had ideas with regard to extracting and filling which are shared in common with a great many in this country, including medical practitioners, and not a dentists, for in his reply he goes on to say that the intention few of the bill was not to interfere with such simple operations as extracting or stopping teeth, which did not require any extended period of study, being performed by chemists. Certain things were allowed and no penalties attached, yet they were to be discouraged. The object of the Bill was not to prevent such simple operations as he had referred to being performed upon anyone who liked to submit himself to the same, but to prevent a person holding himself out to the public as being especially qualified as a dentist when he was not so qualified.

There is a bit of irony in the allusion "any-one who liked to submit himself of the same." In England apparently they make a distinction between those who are *authorized* to practice dentistry, and those who are *allowed* to practice the same. The former have a right to collect a fee, the latter at the mercy of the patient's generosity.

The solicitor in this case took practical ground, when he said that "the proper test would be the candidate's fitness to perform dental operations, no matter where or by what means he gained such knowledge," and certainly this is a common sense view of the matter. The anomaly of the Bill was that while it permitted the druggists to do these things, it rendered them liable to a fine if they advertised or exposed a sign.

English dentistry as a science is outstripping us, but the practice is, judging from these facts, hardly up to ours.

